# REDIportal: toward an integrated view of the A-to-I editing

**DOI:** 10.1093/nar/gkae1083

**Published:** 2024-11-26

**Authors:** Pietro D’Addabbo, Roni Cohen-Fultheim, Itamar Twersky, Adriano Fonzino, Domenico Alessandro Silvestris, Ananth Prakash, Pietro Luca Mazzacuva, Juan Antonio Vizcaino, Andrew Green, Blake Sweeney, Andy Yates, Yvonne Lussi, Jie Luo, Maria-Jesus Martin, Eli Eisenberg, Erez Y Levanon, Graziano Pesole, Ernesto Picardi

**Affiliations:** Department of Biosciences, Biotechnologies and Environment, University of Bari Aldo Moro, via Orabona 4, 70125, Bari, Italy; Institute of Nanotechnology and Advanced Materials, Bar-Ilan University, Ramat Gan, 52900, Israel; Mina and Everard Goodman Faculty of Life Sciences, Bar-Ilan University, Ramat Gan, 52900, Israel; Institute of Nanotechnology and Advanced Materials, Bar-Ilan University, Ramat Gan, 52900, Israel; Mina and Everard Goodman Faculty of Life Sciences, Bar-Ilan University, Ramat Gan, 52900, Israel; Department of Biosciences, Biotechnologies and Environment, University of Bari Aldo Moro, via Orabona 4, 70125, Bari, Italy; Department of Biosciences, Biotechnologies and Environment, University of Bari Aldo Moro, via Orabona 4, 70125, Bari, Italy; European Molecular Biology Laboratory, European Bioinformatics Institute (EMBL-EBI), Wellcome Genome Campus, Hinxton CB10 1SD, UK; Institute of Biomembranes, Bioenergetics and Molecular Biotechnology, National Research Council, via Amendola 122/O, 70126, Bari, Italy; European Molecular Biology Laboratory, European Bioinformatics Institute (EMBL-EBI), Wellcome Genome Campus, Hinxton CB10 1SD, UK; European Molecular Biology Laboratory, European Bioinformatics Institute (EMBL-EBI), Wellcome Genome Campus, Hinxton CB10 1SD, UK; European Molecular Biology Laboratory, European Bioinformatics Institute (EMBL-EBI), Wellcome Genome Campus, Hinxton CB10 1SD, UK; European Molecular Biology Laboratory, European Bioinformatics Institute (EMBL-EBI), Wellcome Genome Campus, Hinxton CB10 1SD, UK; European Molecular Biology Laboratory, European Bioinformatics Institute (EMBL-EBI), Wellcome Genome Campus, Hinxton CB10 1SD, UK; European Molecular Biology Laboratory, European Bioinformatics Institute (EMBL-EBI), Wellcome Genome Campus, Hinxton CB10 1SD, UK; European Molecular Biology Laboratory, European Bioinformatics Institute (EMBL-EBI), Wellcome Genome Campus, Hinxton CB10 1SD, UK; School of Physics and Astronomy, Tel Aviv University, Tel Aviv, 699781, Israel; Institute of Nanotechnology and Advanced Materials, Bar-Ilan University, Ramat Gan, 52900, Israel; Mina and Everard Goodman Faculty of Life Sciences, Bar-Ilan University, Ramat Gan, 52900, Israel; Department of Biosciences, Biotechnologies and Environment, University of Bari Aldo Moro, via Orabona 4, 70125, Bari, Italy; Institute of Biomembranes, Bioenergetics and Molecular Biotechnology, National Research Council, via Amendola 122/O, 70126, Bari, Italy; Department of Biosciences, Biotechnologies and Environment, University of Bari Aldo Moro, via Orabona 4, 70125, Bari, Italy; Institute of Biomembranes, Bioenergetics and Molecular Biotechnology, National Research Council, via Amendola 122/O, 70126, Bari, Italy

## Abstract

A-to-I RNA editing is the most common non-transient epitranscriptome modification. It plays several roles in human physiology and has been linked to several disorders. Large-scale deep transcriptome sequencing has fostered the characterization of A-to-I editing at the single nucleotide level and the development of dedicated computational resources. REDIportal is a unique and specialized database collecting ∼16 million of putative A-to-I editing sites designed to face the current challenges of epitranscriptomics. Its running version has been enriched with sites from the TCGA project (using data from 31 studies). REDIportal provides an accurate, sustainable and accessible tool enriched with interconnections with widespread ELIXIR core resources such as Ensembl, RNAcentral, UniProt and PRIDE. Additionally, REDIportal now includes information regarding RNA editing in putative double-stranded RNAs, relevant for the immune-related roles of editing, as well as an extended catalog of recoding events. Finally, we report a reliability score per site calculated using a deep learning model trained using a huge collection of positive and negative instances. REDIportal is available at http://srv00.recas.ba.infn.it/atlas/.

## Graphical Abstract

**Graphical Abstract:**
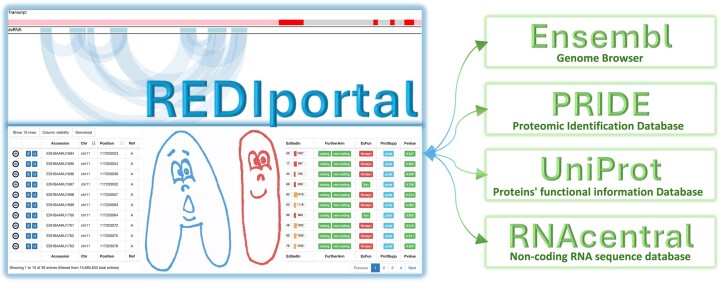

## Introduction

Epitranscriptome modifications are emerging as critical factors in fine-tuning gene expression and regulation (1,2). Among them, A-to-I RNA editing by ADAR (adenosine deaminase acting on RNA) enzymes is pervasive in eukaryotic transcriptomes (3–5). In humans, where this phenomenon has been investigated for a while, it has been shown to play several key biological roles depending on its location in the target RNA (i.e. Coding Sequence (CDS), intron or Untranslated Regions (UTRs)) (6). A-to-I editing can modify the basic properties of neurotransmitter receptors (7,8), modulate alternative splicing (9,10), influence the biogenesis of circRNAs and microRNAs (11–13) or tune their effects acting on their target RNAs (14). The majority of known events occur in repetitive regions and mostly in double-stranded RNAs (dsRNAs) formed by Alu elements in opposite orientations (15–17). A-to-I editing in dsRNAs is pivotal in cytosolic innate immunity by suppressing type I interferon signaling (18,19). On the contrary, only a small fraction of A-to-I conversions has been described in protein-coding regions. These can lead to amino acid changes (recoding sites) (20). Recoding sites are extensively investigated due to the potential for downstream functional effects, and their deregulation was associated with different human disorders including but not limited to neurological, neurodegenerative, autoimmune, cardiovascular diseases and cancer (21–23).

Large-scale deep transcriptome sequencing has fostered the characterization of A-to-I editing at the single nucleotide level and several computational tools for its accurate detection have been released (24–27). Harnessing >9000 RNA sequencing (RNAseq) experiments from the GTEx project and a rigorous bioinformatics protocol based on our REDItools package (25,28), we collected ∼16 million of A-to-I events across 31 tissues and 54 body sites of 549 individuals, populating the REDIportal database (5). Our knowledgebase tool represents a unique and comprehensive A-to-I catalog and is becoming a reference resource for the scientific community and all researchers involved in the epitranscriptome field. REDIportal is queried to investigate RNA editing in personal datasets or downloaded for developing novel computational resources or serving as a benchmark. For instance, several databases such as REIA (29), TCEA (30), CAeditome (31) and GPedit (32) are based on REDIportal annotations. In the current year, its main webpage has been viewed >23 000 times with a mean of ∼2300 views per month (∼77% more than last year) (source Google Analytics—https://analytics.google.com).

Investigating RNA editing may facilitate a better understanding of yet unknown molecular aspects of this non-transient epitranscriptome modification, unveil associations with human disorders, or discover biomarkers with a prognostic or diagnostic value (33). Understanding editability properties is also crucial for designing therapeutic interventions based on site-directed RNA editing approaches, such as correcting disease-causing mutations (34). From a computational point of view, large collections of A-to-I events can be exploited to feed machine and deep learning algorithms for comprehensive editing profiling without the need for specific genomic data or tedious filtering steps implemented in cumbersome bioinformatics pipelines. To improve the investigation of RNA editing and face the current challenges of epitranscriptomics, we have updated REDIportal adding new data and functionalities, maintaining the same layout to leave unchanged the user experience. This third major REDIportal release adds A-to-I changes from >9000 TCGA RNAseqs of primary tumors (covering 31 studies) (35). Available cancer editing databases, such as TCEA (30), CAeditome (31), GPedit (32) or REIA (29), are all built using the first or second REDIportal release. Our new release improves on these resources and includes the AEI (Alu editing index) (36) and REI (recoding index) (21) indices, two well-established metrics to quantify RNA editing activity globally or at recoding sites only, as well as expression values for the ADAR genes (37). Given the relevance of A-to-I editing in innate immunity (6,15,38,39), REDIportal implements a novel module to study ADAR-mediated editing in putative dsRNAs by providing dsRNA indices per transcript calculated likewise the AEI index. To provide an accurate, sustainable and accessible database, this new release of REDIportal is interconnected with Ensembl (40), UniProt (41), RNAcentral (42) and PRIDE (43), which are widespread and internationally recognized ELIXIR core data resources, part of the European infrastructure for life sciences.

This new REDIportal release is also enriched with an increased portfolio of recoding sites detected in GTEx data by a recent ad hoc computational procedure (20). Finally, REDIportal events have been associated with a reliability score calculated using our REDInet (https://github.com/BioinfoUNIBA/REDInet) algorithm implementing a deep learning model trained using a huge collection of positive and negative examples (44).

All the updates and database improvements described in this manuscript are mainly related to human data. REDIportal is still freely available at http://srv00.recas.ba.infn.it/atlas/index.html.

## Data collection and processing

### Data collection

TCGA RNAseq data comprising 9683 experiments from primary tumors across 31 studies (and 24 disease types) were downloaded from the GDC Data Portal (https://portal.gdc.cancer.gov/) (dbGaP accession number phs000178) in BAM format using the GDC Data Transfer Tool Client (https://gdc.cancer.gov/access-data/gdc-data-transfer-tool). Since BAM files were processed by aligning reads onto the hg38/GRCh38 human reference genome by STAR (45) and using Gencode annotations, only a preliminary filtering step was performed to retain uniquely mapped reads. All alignments were saved in sorted and indexed BAM files using SAMtools (version 1.9) (46).

Recoding editing sites in GTEx samples, detected using the computational strategy described in (20) implementing stringent filters for protein-coding regions, were downloaded from the publisher website and merged into REDIportal. Resulting A-to-I editing sites were checked to rule out duplicates and positions showing strand biases. The non-redundant list of 15 680 833 bona fide A-to-I editing events was annotated using ANNOVAR and the following updated databases: (i) RepeatMasker containing known repetitive elements; (ii) dbSNP (version 155) collecting genomic single nucleotide polymorphisms (35); (iii) Gencode (v45) (31), RefSeq (36) and UCSC (Genome Browser database) (37) storing gene and transcript annotations; and (iv) PhastCons providing conservation scores across 100 species.

For each site, a unique alphanumeric accession number was generated, allowing the recovery of editing sites independently from the genome assembly and database version, promoting interoperability. To cover annotated sites and those that may originate from future releases, each accession number is composed of eight alphanumeric characters prefixed with ED (for ‘EDiting’) and an organism code (of at least two letters such as HS for human or MM for mouse), and followed by four upper-case letters and four digits, allowing >4.5 billion combinations per organism.

A-to-I sites collected in REDIportal have been detected using an empirical computational protocol based on our REDItools software (25,28). It identifies editing events according to specific filters that are tuned depending on the RNAseq features (i.e. base quality, mean depth and so on) (28). To improve the reliability of sites collected in REDIportal, we have now introduced an aggregated *P*-value calculated by our REDInet algorithm (https://github.com/BioinfoUNIBA/REDInet). It is based on a deep learning model trained using a balanced dataset comprising >9 million positives and negatives. For each site of a given RNAseq, REDInet explores its surrounding bases and assigns an editing *P*-value. REDInet was applied to all GTEx RNAseq data and resulting *P*-values, aggregated per tissue and body site, were included in the main REDIportal table. REDInet *P*-values have been calculated for human data only since no large A-to-I collections for training are yet available for other organisms.

### A-to-I editing detection in TCGA data and editing metrics

RNA variants per sample were called using an HPC-optimized version of our REDItools package (47) and stored in tabular format. REDItools was launched on individual BAM files with non-stringent parameters to provide a comprehensive catalog of all putative and non-putative RNA variants. Output tables were gzipped and indexed by tabix. Known A-to-I editing sites from the latest REDIportal list were extracted from indexed REDItools tables by a custom Python script. *De novo* events were not included in the current release due to computational restrictions. The detection of novel editing sites, indeed, requires stringent and time-consuming filtering steps to take into account somatic changes and regions with altered copy numbers, other than extensive quality checks.

**Figure 1.**
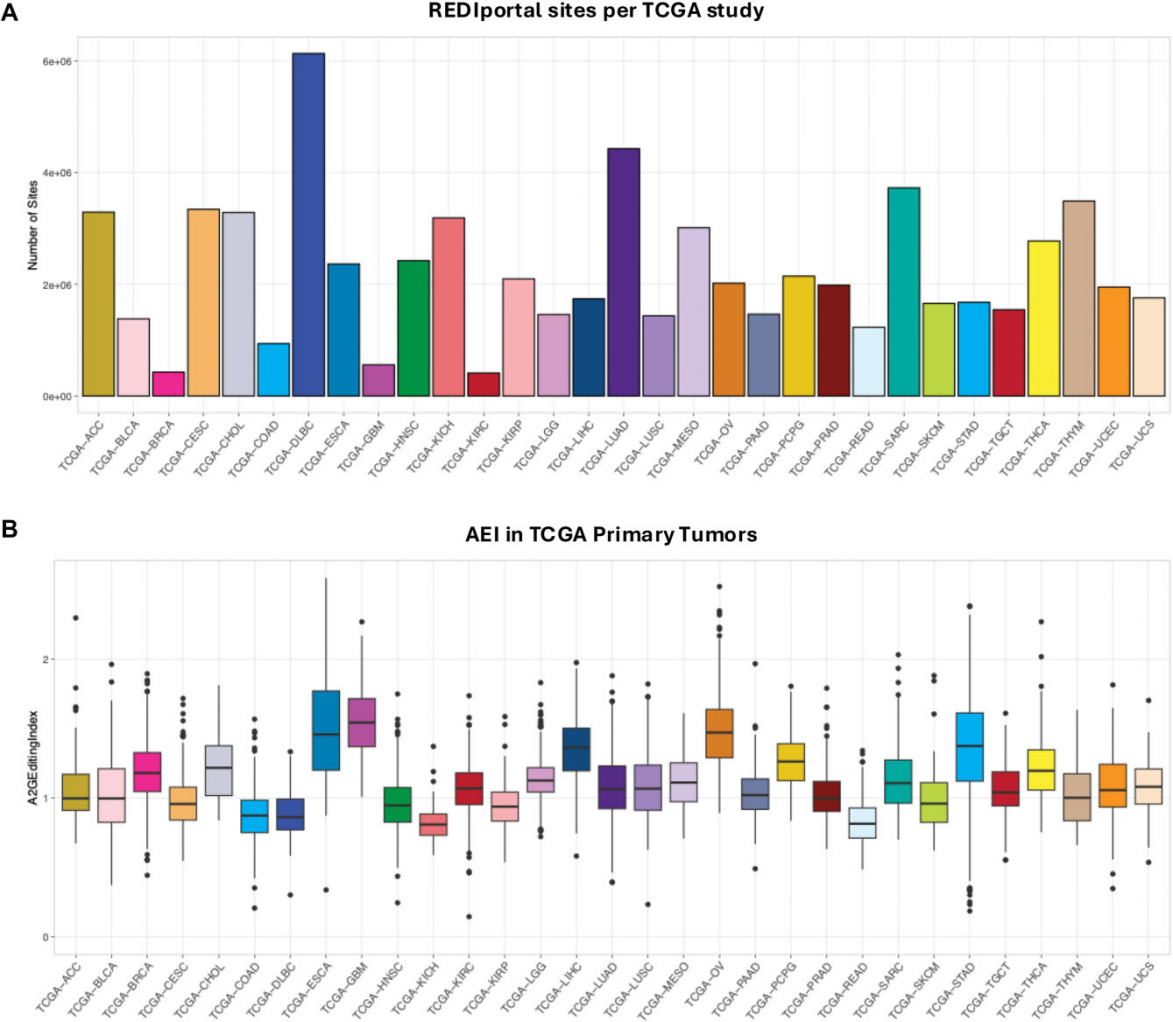
(**A**) Distribution of REDIportal sites across 31 TCGA studies. (**B**) Box plot of AEI per TCGA study.

On average 2 166 001 known editing sites appeared edited in TCGA studies with sufficient coverage (>10 reads). The highest number of events was found in TCGA-DLBC with samples from the lymphoid neoplasm diffuse large B-cell lymphoma (Figure 1A). In contrast, TCGA-KIRC with samples from kidney renal clear cell carcinoma contained the lowest number of editing sites (Figure 1A).

In contrast with the available cancer editing databases, REDIportal includes the AEI and REI metrics to quantify the RNA editing activity globally or at recoding sites, respectively. The AEI, defined as the weighted average of editing events occurring at all adenosines within Alu elements, was calculated using the RNAEditingIndexer program (https://github.com/a2iEditing/RNAEditingIndexer) (36). The REI index, instead, was computed as the weighted average over all known recoding sites, as described in (21) and implemented in (37) (https://github.com/BioinfoUNIBA/QEdit). The AEI index is particularly relevant, allowing users to select samples and tumor types according to the RNA editing activity (Figure 1B) and, thus, focus only on events characterized in a given cancer study. The REI index is also as important as AEI in cancer genomics because it allows the identification of tumor studies with an altered recoding activity that could lead to cancer-specific biomarkers. In GBM, for instance, the study of the recoding activity allowed the finding of the COG3 I/V as an eligible site for personalized targeted gene therapy (21).

### RNA editing in dsRNAs

ADAR-based editing is pivotal in suppressing innate immune interferon responses triggered by cellular dsRNAs through the MDA5–MAVS axis (18,19). Consequently, investigating RNA editing in dsRNAs can offer valuable insights into the study of immunogenic dsRNAs (6,15) and deciphering the roles of dsRNA editing in inflammatory and autoimmune diseases and cancer, fostering the design and development of novel therapeutic approaches.

From a computational point of view, the detection of dsRNA editing is challenging because real RNA secondary structures remain elusive. Nonetheless, putative dsRNAs could be identified following an approach proposed by Barak *et al.* (48). Using the latest RNAseq curated annotations (downloaded from UCSC in May 2024), we selected non-overlapping gene loci and, for each locus, we extracted the longest mature transcript. Each transcript was aligned with itself by BLAST and plus/minus hits were extracted as putative dsRNAs. For each GTEx sample, we used REDItools to call A-to-I editing falling in the collection of dsRNAs (using those with length >70 bases and identity >70%), filtering out sites annotated in dbSNP (version 155). Individual dsRNAs were annotated using RepeatMasker (downloaded from UCSC in May 2024). To quantify the editing activity per dsRNA or the dsRNA editing activity per transcript, we calculated a dsRNA index likewise the AEI index. In particular, the dsRNA index is defined as the weighted average of editing events occurring in all adenosines within dsRNAs. Since most dsRNAs are due to Alu elements in opposite orientations, the dsRNA index is a proxy of the AEI index but provides insights at the transcript level.

In REDIportal, dsRNA annotations and dsRNA indices for all GTEx samples are stored in a dedicated MySQL table, queryable by gene name.

### REDIportal in the ELIXIR ecosystem

**Figure 2.**
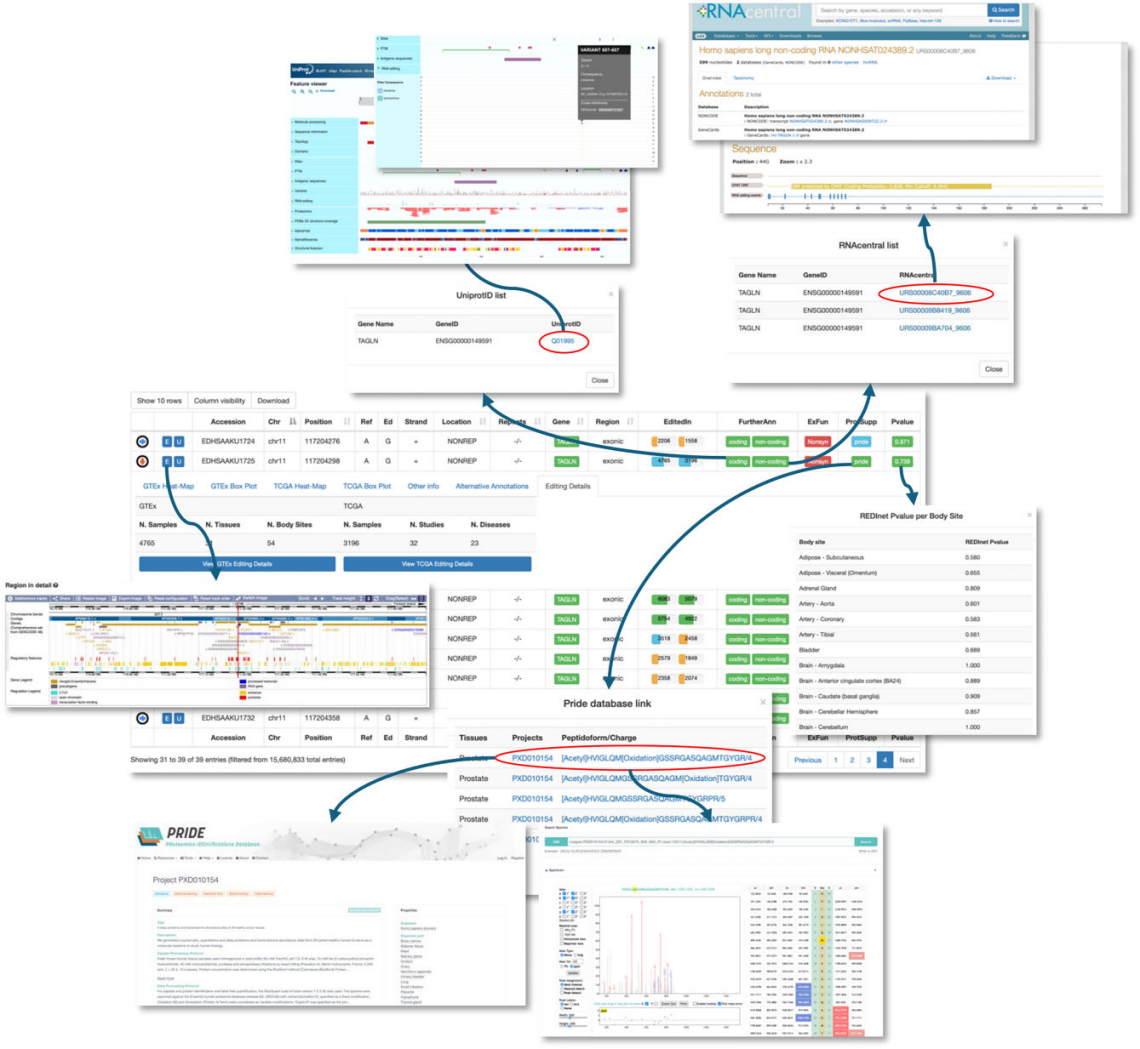
An overview of data integrated in REDIportal. Searched RNA editing sites are shown in sortable tables and for each genomic position several details are provided. In REDIportal v3, editing sites are interconnected to ELIXIR core data resources. If a given site falls in a protein-coding region, a link to UniProt database is provided. Proteomic support (if any) by the PRIDE database is also included by dedicated links. For sites in ncRNAs, specific links to RNAcentral have been added. Users can also browse editing sites in their genomic context by Ensembl. On the contrary, REDIportal sites can also be reached from UniProt, RNAcentral, Ensembl and PRIDE. Circles underscore REDIportal links to ELIXIR core resources. Arrows point to REDIportal popups and ELIXIR core resources.

ELIXIR is the European life sciences research infrastructure aimed to enable researchers to access and analyze life science data, to improve the value and impact of life science research on public health, the environment and the economy (49) (https://elixir-europe.org/). It involves scientists from 23 European countries and over 250 research institutes. ELIXIR coordinates and develops a large number of bioinformatics resources across Europe including well-established tools of fundamental importance to the wider lifescience community (49). Since epitranscriptomics is yet an under-represented field in the ELIXIR community, and REDIportal is in the ELIXIR portfolio of services (https://elixir-europe.org/services/list), our knowledge base tool has been strengthened through interconnections to existing ELIXIR core data resources such as Ensembl, UniProt, RNAcentral and PRIDE (Figure 2). Through links to Ensembl (40), users can browse editing sites in their genomic context, complementing the JBrowse module already running in REDIportal (Figure 2). On the other end, Ensembl users can now visualize RNA editing tracks and bind to REDIportal for further details. Non-coding RNAs (ncRNAs) in humans as well as other eukaryotes are critical components of cellular machinery (50,51), and many of them are subjected to RNA editing that could modulate their structure and function (52,53). To facilitate the study of A-to-I editing in ncRNAs, REDIportal annotations have been included in RNAcentral (42), a database of ncRNA sequences that aggregates data from specialized ncRNA resources and provides a single entry point for accessing ncRNA sequences of all ncRNA types from all organisms. In RNAcentral (https://rnacentral.org/), users can search for all ncRNAs with an editing event and visualize editing sites in a specific non-coding context. In REDIportal, instead, users interested in ncRNAs can point to RNAcentral through dedicated hyperlinks (Figure 2). To connect REDIportal data to RNAcentral sequences, RNAcentral intersected the genomic coordinates of all editing events in REDIportal with known coordinates of all ncRNAs. They then mapped the genomic coordinates into coordinates within a sequence and used this to visualize the editing events in a non-coding context. Future work will focus on showing the editing events in our secondary structure viewer as well as within the sequence.

ADAR-mediated editing can occur in protein-coding genes and lead to recoding events, i.e. non-synonymous substitutions that generate novel protein isoforms (6,52). Although small in number, recoding sites are over-represented in transcripts encoding proteins linked to the nervous system function and may have crucial physiological roles (20,22,54). For example, the recoding R/G site in the GRIA2 gene for the glutamate receptor subunit B regulates the desensitization kinetics of the receptor (55), while the Q/R site in the same gene regulates the Ca^2+^permeability of the ion channel and its alteration is related to neuronal death (56).

To better investigate recoding events and their potential functional roles, REDIportal recoding events have been interconnected to UniProt (41) (https://www.uniprot.org/). In particular, all recoding events supported by three annotation databases such as Gencode, RefSeq and UCSC, and showing editing evidence in at least five GTEx individuals and a pooled edited level >1%, were extracted from REDIportal. The resulting sites were mapped on UniProt annotations by the ID mapping tool (https://www.uniprot.org/id-mapping) to detect UniProt accession numbers. Such unique identifiers were included in REDIportal while recoding sites falling in UniProt entries were added in UniProt in the Feature Viewer and handled as a variation track (Figure 2).

Detecting recording sites in large transcriptome datasets is still a challenging task (20). Proteomics data such as those from mass spectrometry experiments can help in identifying peptides containing editing-modified amino acids. The PRIDE database (43) (https://www.ebi.ac.uk/pride/) is the world-leading database for mass spectrometry-based proteomics data. Public proteomics datasets can be harnessed to look at edited positions at the proteome level using a proteogenomics approach. For this purpose, public proteomics datasets were selected and reanalyzed, considering the most reliable edited sites that have been observed at the transcriptome level and annotated at REDIportal. In particular, REDIportal recoding sites used for UniProt mappings were further filtered to remove sites in repeat elements and observed in <10 GTEx samples, yielding a list of 16 184 A-to-I edited sites. Overall, 11 public proteomics datasets from the PRIDE database (see Supplementary Table S1 for the list of datasets) were reanalyzed comprising 2001 human samples. Samples were searched using MaxQuant (version 2.1.0.0) (57) against a database of peptide sequences generated using the UniProt human proteome (downloaded in May 2023) as the basis. In brief, the protein sequences were *in silico* digested with trypsin to generate >1.2 million peptides. Combinations of ‘edited’ peptide sequences were generated by substituting only one potentially edited amino acid residue onto a native peptide sequence. Finally, native peptide sequences and all combinations of ‘edited’ peptides were pooled together into a target peptide search database. A detailed description of the methodology for generating the target peptide search database can be found in the Supplementary File.

The datasets were reanalyzed considering a statistical threshold of 1% false discovery rate at a peptide level. A total of 24 edited amino acid sites were identified in 22 proteins corresponding to 92 peptide spectrum matches (PSMs). Additionally, after the manual inspection of PSMs of the detected sites, low-quality PSMs were discarded. Overall, 11 peptides with an edited amino acid substitution were kept, which were supported by 48 PSMs. These peptides were included in REDIportal. Universal Spectrum Identifiers (USI) (58) can be used to visualize the source mass spectra corresponding to these peptides, linked from REDIPortal into PRIDE (Supplementary Table S2). Examples of proteins in which edited peptides were identified include Transgelin (TAGLN, UniProt accession: H0YCU9) at amino acid position 132 N>S (chr 11: 117204298, A>G) in prostate and smooth muscle samples (USI: https://www.ebi.ac.uk/pride/archive/usi?usi=mzspec%3APXD010154%3A01323_D01_P013562_S00_N04_R1%3Ascan%3A15878%3A%5BAcetyl%5DHVIGLQM%5BOxidation%5DGSSRGASQAGMTGYGR%2F4) (Figure 2) and Filamin A (FLNA, UniProt accession: H0Y5C6) at amino acid position 281 Q>R (chr X: 154351582, T>C) in vermiform appendix (USI: https://www.ebi.ac.uk/pride/archive/usi?usi=mzspec:PXD015079:190319-Labrie-3454-APP:scan:10364:LTVSSLR/2).

Detecting peptides changed by RNA editing is quite challenging and computationally intensive. As more datasets are reanalyzed, we hope it will be possible to provide additional results in future REDIportal releases.

## Database content and web interface

### Update of the database content

REDIportal collects 15 680 833 sites from 9642 human GTEx RNAseq samples (across 31 tissues and 54 body sites) and 9683 human TCGA RNAseq samples (across 31 studies and 24 disease types). Although human-oriented, REDIportal collects also 107 095 sites from mouse RNAseq samples, covering three different tissues.

REDIportal is organized in four MySQL tables. MySQL TABLE1 includes individual sites, their annotations and cross-links to external resources (Ensembl, UniProt, RNAcentral and PRIDE). MySQL TABLE2 stores RNA editing levels per RNAseq, while statistics and RNA editing metrics (AEI and REI indices) per GTEx and TCGA sample are allocated in MySQL TABLE3. RNA editing data for dsRNAs are instead included in the novel MySQL TABLE4. It comprises genomic coordinates of dsRNAs and dsRNA indices.

### Update of the web interface

REDIportal inherits the layout from the previous release to leave the user experience unchanged. All web pages are developed in Bootstrap, CSS and JavaScript. Server-side operations to query MySQL tables and retrieve data are performed in Python and require MySQLdb (https://pypi.python.org/pypi/MySQL-python/1.2.5) and mxTextTools (http://www.egenix.com/products/python/mxBase/mxTextTools/) as external modules for MySQL connections and high-performance text manipulation, respectively.

**Figure 3.**
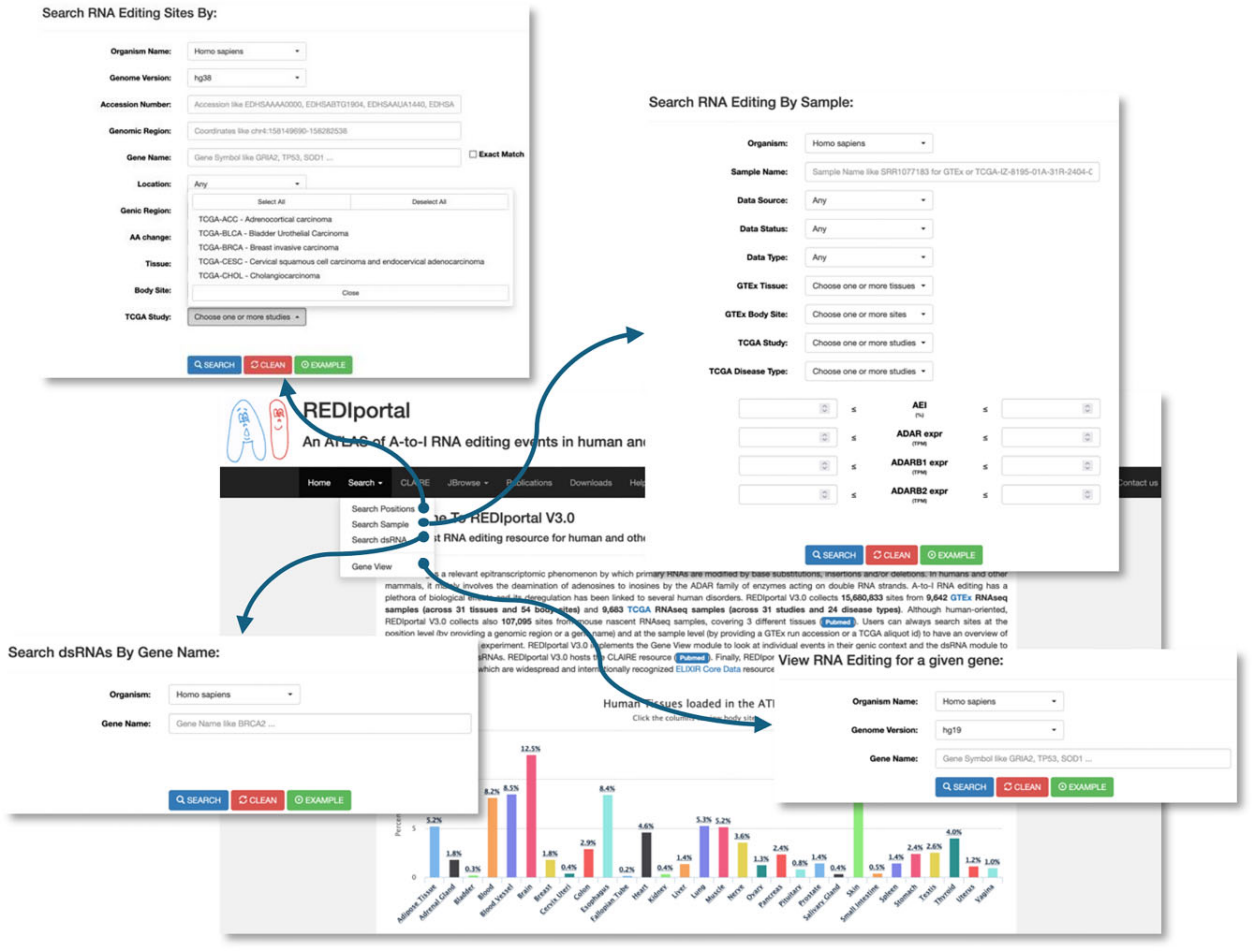
Searching the REDiportal database. In REDIportal v3, RNA editing data can be queried by four dedicated search pages (indicated by arrows) depending on the required details. As a novelty, users can now explore RNA editing in dsRNAs.

The REDIportal search dropdown menu now includes four pages: (i) ‘Search Positions’ to perform queries at the position level by providing a genomic region (in the format chr:start-end) or a gene symbol. A specific GTEx tissue, GTEx body site, TCGA study or a combination of them can also be selected. (ii) ‘Search Sample’ to interrogate one or more GTEx or TCGA samples by providing a string of run accessions (for GTEx) or aliquots IDs (for TCGA). Samples can also be selected according to AEI or ADAR expression values. (iii) ‘Search dsRNA’ to retrieve editing in dsRNAs by providing a gene name. (iv) ‘Gene View’ to visualize RNA editing events in their genomic context (Figure 3).

The ‘Search Positions’ allows now the retrieval of A-to-I sites in TCGA studies while ‘Search Sample’ enables also the browsing of TCGA samples and includes five panels per sample: (i) ‘Genomics Facts’; (ii) ‘Base Distribution’; (iii) ‘RNA Editing Indices’; (iv) ‘RNA Editing Levels’; and (v) ‘Transcriptome Coverage’. The button ‘REI details’ now allows the browsing of >2000 known recoding events.

RNA editing sites retrieved by the ‘Search Positions’ page are shown in dynamic and sortable tables. For each human A-to-I site, REDIportal now shows the following info: (i) the unique accession number; (ii) two links to Ensembl and UCSC genome browsers; (iii) the genomic position; (iv) the reference and edited nucleotide; (v) the strand; (vi) the editing location; (vii) the repeated element (if any); (viii) the gene symbol according to Gencode v45 linked to GeneCards; (ix) the genic region; (x) the number of edited samples in GTEx and TCGA projects; (xi) links to UniProt (if in protein-coding gene) or RNAcentral (if in ncRNA); (xii) the potential amino acid change; (xiii) the PRIDE proteomic support with links to the original dataset and the mass spectrum of the detected edited peptide; and (xiv) the aggregated REDInet *P*-value calculated as the mean of *P*-values across all body sites (Figure 2).

Extra info per site is located in child rows and now consists of seven panels: (i–ii) an interactive heat map and box plot to look at RNA editing levels across GTEx body sites; (ii–iii) an interactive heat map and box plot for editing levels in TCGA studies; (iv) alternative gene/transcript annotations according to RefSeq and UCSC databases; (v) editing details with the number of edited samples in GTEx and TCGA, and links to the corresponding RNA editing levels; and (vi) other info containing the Ensembl gene ID, the gene type (according to Gencode), the phastCons score across 100 species, the dbSNP accession (if any) and a flag indicating whether the site was already known in previous editing databases [including RADAR (59) and DARNED (60)] (Figure 2).

**Figure 4.**
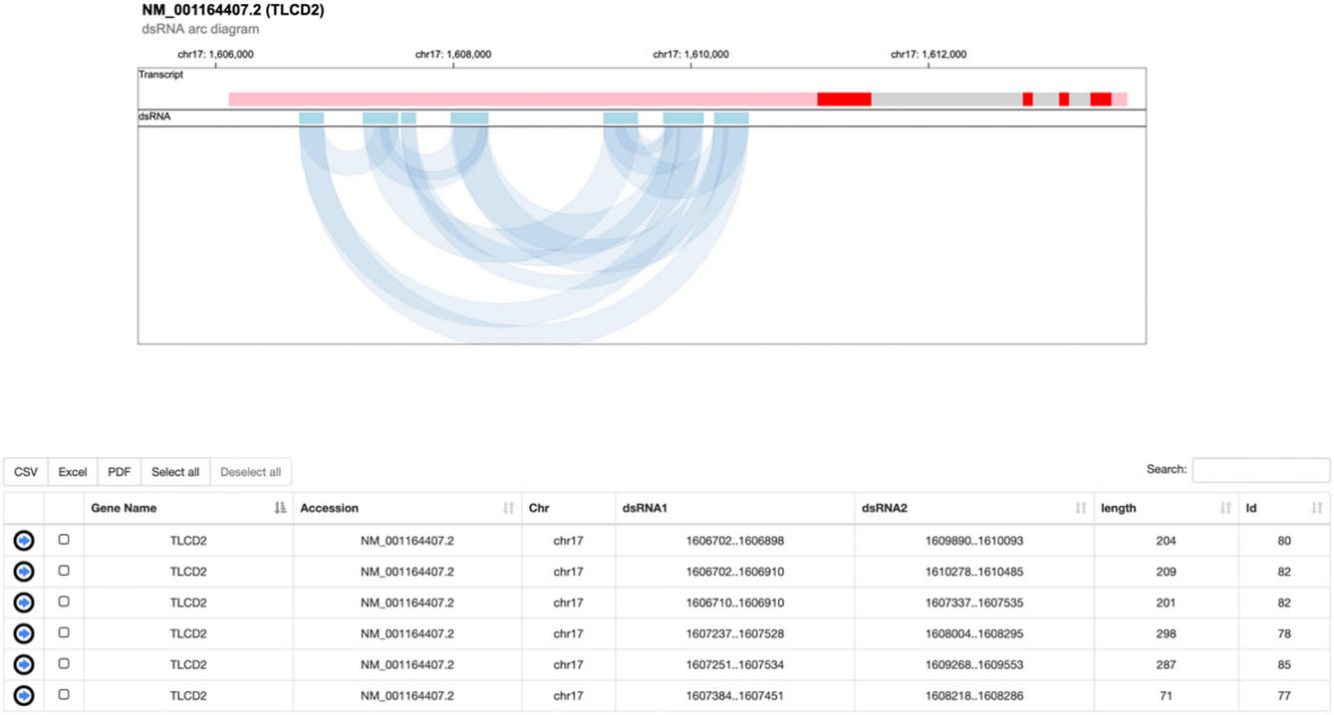
DsRNAs in REDIportal. The new REDIportal version allow the investigation of dsRNAs reported along the transcript structure by arc diagrams using gosling. A table comprising dsRNA coordinates and dsRNA indices is also showed.

Double-stranded RNAs can be retrieved through the ‘Search dsRNA’ page and be visualized as arc diagrams in their transcript context by gosling (61) (http://gosling-lang.org/). A table reporting dsRNAs genomic coordinates as well as dsRNA editing indices is also displayed (Figure 4).

## Conclusion and future plans

A-to-I RNA editing is a functionally relevant epitranscriptome modification that plays crucial roles in humans and other organisms (6,62). To foster its investigation, we developed REDIportal (4,5), a specialized database comprising the largest collection of A-to-I changes. Its third release, mainly human-oriented, comprises ∼16 million events in >18 000 RNAseqs from GTEx and TCGA projects. REDIportal has now been included in the ELIXIR ecosystem by interconnections with widespread core resources such as Ensembl, RNAcentral, UniProt and PRIDE, and enables the study of edited dsRNAs.

To face the current challenges of epitranscriptomics, we plan to extend the repertoire of editing sites with *de novo* events from TCGA samples [facilitating the prediction of cancer neo-antigenes (63,64)] and large transcriptome data produced by several consortia. Although REDIportal is mainly human-oriented, in the next releases we plan to increase the number of organisms and adapt the current output layout also to sites from other organisms whose data are already collected in REDIportal (such as mouse).

New sequencing technologies such as direct RNA sequencing by Oxford Nanopore Technologies (ONT) are now available to profile RNA editing and RNA modifications in general (65–67). They hold the promise to detect inosine and modified adenosines at the transcript level (66,68). We plan to update REDIportal with a dedicated module to deal with editing annotations from ONT data. Our future ambition is to collect also modified adenosines (m^6^A or m^1^A) to investigate the interplay between A-to-I editing and RNA modifications and provide an overview of epitranscriptome changes at the single and transcript level. Since ONT also allows the accurate detection of C-to-U editing events (69), we plan to include them in a future REDIportal release.

## Supplementary Material

gkae1083_Supplemental_Files

## Data Availability

REDIportal is available through the following web page: http://srv00.recas.ba.infn.it/atlas/. The full list of sites can be downloaded from the REDIportal download page (http://srv00.recas.ba.infn.it/atlas/download.html). REDItools, used to profile RNA variants in RNAseq data, is available at the following GitHub repository: https://github.com/BioinfoUNIBA/REDItools and https://doi.org/10.5281/zenodo.13981272.
