# Supplementary material for: REDIportal: toward an integrated view of the A-to-I editing
Source: Nucleic Acids Res. 2024 Nov 26;53(D1):D233–42. doi: 10.1093/nar/gkae1083 (PMC11701558; doi:10.1093/nar/gkae1083)
Supplement: gkae1083_Supplemental_Files [file gkae1083_supplemental_files.zip › REDIportal Supplementary File.pdf]

Supplementary Methods and Figures

REDIportal: towards an integrated view of the A-to-I editing

Pietro D'Addabbo[1], Roni Cohen-Fultheim[2,3,§], Itamar Twersky[2,3,§], Adriano Fonzino[1], Domenico Alessandro Silvestris[1], Ananth Prakash[4], Pietro Luca Mazzacuva[5], Juan Antonio Vizcaino[4], Andrew Green[4], Blake Sweeney[4], Andy Yates[4], Yvonne Lussi[4], Jie Luo[4], Maria-Jesus Martin[4], Eli Eisenberg[6], Erez Y. Levanon[2,3], Graziano Pesole[1,5], Ernesto Picardi[1,5,*].

[1]Department of Biosciences, Biotechnologies and Environment, University of Bari Aldo Moro.
[2]Institute of Nanotechnology and Advanced Materials, Bar-Ilan University, Ramat Gan, Israel.
[3]Mina and Everard Goodman Faculty of Life Sciences, Bar-Ilan University, Ramat Gan, Israel.
[4]European Molecular Biology Laboratory, European Bioinformatics Institute (EMBL-EBI), Wellcome Genome Campus, Hinxton, CB10 1SD, United Kingdom.
[5]Institute of Biomembranes, Bioenergetics and Molecular Biotechnology, National Research Council.
[6]School of Physics and Astronomy, Tel Aviv University, Tel Aviv 69978, Israel.

Generating a target search peptide database

A-to-I editing-mediated amino acid substitution sites from REDIportal were used to generate the target search database for proteomics data analysis. The REDIPortal editing-mediated amino acid substitution sites were filtered to remove (i) synonymous substitutions, (ii) substitutions within repeat elements (ALU/SINE, etc.), and (iii) observed in less than 10 samples, resulting in 16,184 A-to-I editing-mediated substitution sites from 2,692 proteins. Supplementary figure 1B shows the distributions of A-to-I editing-mediated substitution sites on proteins. Given that a large number of proteins have more than 30 A-to-I editing-mediated substitution sites on them, generating a protein sequence database, with all possible variant protein sequence combinations, will result in an extremely large number of protein sequences and would not feasible for database searching (as part of the proteomics data analysis). Therefore a peptide database was created.
To keep the number of peptides to a feasible number for database search, the compact UniProt human 'one protein sequence per gene' protein sequence database (n=20,593 sequences, downloaded in May 2023) was used to generate the target peptide search database. The protein identifiers from REDIportal filtered

editing-mediated substitution sites were first compared to the proteins in the ‘one protein sequence per gene’ protein sequence database, which had only 492 (18.3%) protein identifiers in common, since the ‘one protein sequence per gene’ database does not contain isoforms. The remaining proteins from REDIPortal sequences were taken from the UniProt human ‘reference proteome with isoforms’ database (n=104,573 sequences, downloaded May 2024). Of the 2,200 proteins that were not present in the ‘one protein sequence per gene set’ database, only 1,396 (36.4%) proteins were found in the ‘reference proteome with isoforms’ database, the remaining 804 (36.5%) proteins identifiers were ‘lost’ due to them being deprecated or merged with another protein accession in the subsequent release of UniProt database.

The 1,396 protein sequences were appended to the ‘one protein sequence per gene’ protein sequence database bringing the total number of protein sequences to 21,989. Using the new ‘appended’ protein sequence database, protein sequences were first *in-silico* digested at trypsin cleavage sites allowing 2 missed cleavage sites resulting in overlapping peptide sequences. Tryptic peptides with length between 7 and 70 amino acids long were considered for downstream analysis (Supplementary figure 1C). From the distribution of editing-mediated amino acid substitution sites on tryptic peptides (Supplementary figure 1D) only 0.87% (n=22 peptides) of the total peptides had 21 or more A-to-I mediated amino acid substitution sites on them. To reduce computational complexity, peptides with 21 or more A-to-I mediated amino acid substitution sites on them were removed.

The amino acid coordinates from A-to-I editing-mediated sites in REDIportal were used to substitute corresponding amino acids onto the peptide sequences, including generating two versions of a peptide where, due to codon changes, the same amino acid position was substituted. Each peptide sequence was given an unique identifier, which in the following order, comprises: (i) UniProt accession, (ii) sequential number of tryptic peptide on the protein sequence (Peptide_), (iii) position on the peptide sequence where the amino acids are substituted if A-to-I editing takes place (Editpos:) (native unedited peptide sequences have *Editpos* value ‘NA’) and (iv) variant number of the peptide (var:), which can distinguish it when the same amino acid position was edited because of a change in codon, example: tr|E9PLD2|E9PLD2_HUMAN|Peptide_17_Editpos:5_var:3 and tr|E9PLD2|E9PLD2_HUMAN|Peptide_17_Editpos:5_var:5). This resulted in a search database with a total of 1,212,450 peptides comprising both unmodified native peptide sequences and ‘edited’ peptides. Supplementary figure 2 shows the database creation pipeline. The peptide search database and table of edited peptides can be found here: https://ftp.ebi.ac.uk/pub/databases/pride/resources/AtoI/1mod_per_peptide_plus/

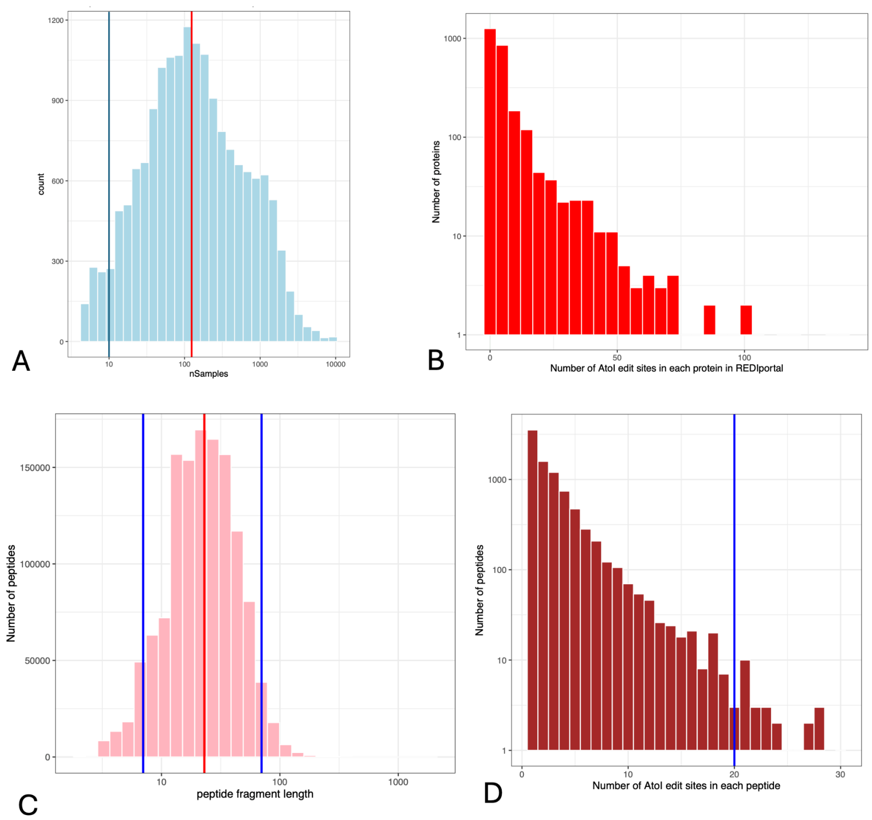

Supplementary Figure 1. (A) Distribution of A-to-I editing-mediated amino acid sites from the REDIportal database across a number of samples. Red line indicates the median and the blue line shows the filter (n=10 samples). **(B)** Distribution of A-to-I editing-mediated amino acid sites from the REDIportal database in each protein. **(C)** Distribution of tryptic peptide lengths. The red line shows the median values, blue lines indicate the filter between 7 and 70 amino acid long peptides. **(D)** Distribution of A-to-I editing-mediated amino acid sites in each tryptic peptide. The blue line indicates the filter threshold where a tryptic peptide has more than 20 A-to-I sites.

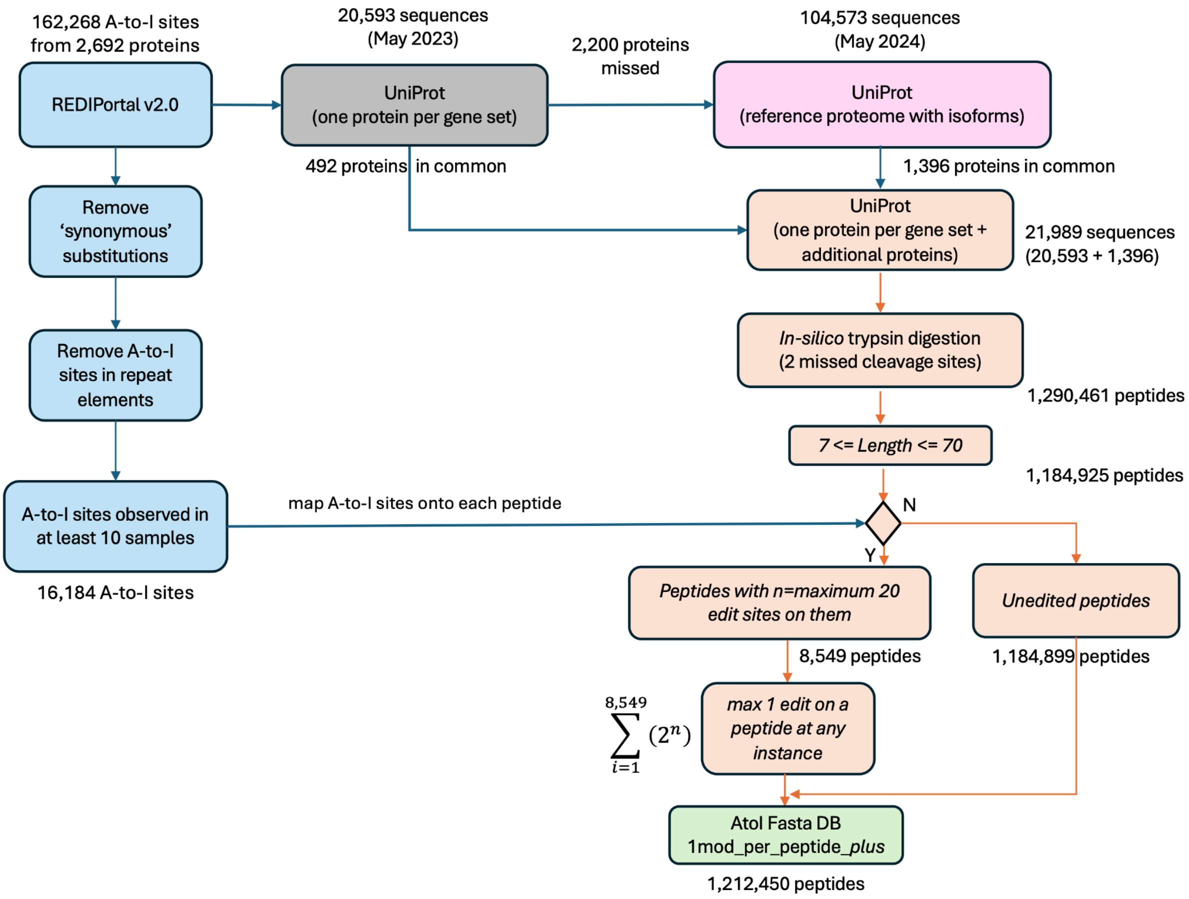

Supplementary Figure 2. Pipeline showing the generation of A-to-I editing-mediated peptide search database3.

Searching proteomics datasets

Public mass spectrometry (MS) proteomics datasets were downloaded from the PRIDE database. Overall, 11 human baseline proteomics datasets representing 31 healthy tissue samples (Supplementary table 1) were analysed using MaxQuant (version 2.1.0.0) (1) on a high performance Linux computing cluster. The input parameters for each dataset such as MS1 and MS2 tolerances, digestive enzymes, fixed and variable modifications were set as described in their respective publications together with two missed cleavage sites. PSM (Peptide Spectrum Match) and peptide FDR (False Discovery Rate) levels were set at 1%. Other MaxQuant parameter settings were left as default: maximum number of modifications per peptide: 5, minimum peptide length: 7, maximum peptide mass: 4,600 Da. For the "match between runs" option, the minimum match time window was set to 0.7 seconds and the minimum retention time alignment window was set to 20 seconds. The peptide database describe above was used as the target search database and the inbuilt MaxQuant contaminant database was used and the decoy database were generated by MaxQuant at the time of the analysis (on-the-fly) by reversing the input database sequences after the respective enzymatic cleavage.

The datasets were run in a multithreading mode with a maximum of 100 threads and 500 GB of RAM per dataset.
The raw output from MaxQuant, along with PSM evidences for identifications of each dataset can be found here: https://ftp.ebi.ac.uk/pub/databases/pride/resources/AtoI/1mod_per_peptide_plus/

**PRIDE dataset**	**Tissues**	**Number of .raw files (samples)**
PXD010154	Adipose tissue, Adrenal gland, Bone marrow, Brain, Pituitary hypophysis, Colon, Duodenum, Esophagus, Fallopian tube oviduct, Gallbladder, Heart, Kidney, Liver, Lung, Lymph node, Ovary, Pancreas, Placenta, Prostate, Rectum, Salivary gland, Small intestine, Smooth muscle, Spleen, Stomach, Testis, Tonsil, Urinary bladder, Uterine endometrium, Vermiform appendix	1,547
PXD005819	Brain	33
PXD006233	Brain	192
PXD012755	Brain	15
PXD000547	Brain	40
PXD000548	Brain	40
PXD004332	Brain	56
PXD010271	Brain, Liver, Ovary, Pancreas	55
PXD008934	Heart	7
PXD020187	Umbilical artery	10
PXD015079	Brain, Vermiform appendix	6

Supplementary Table 1. List of reanalyzed PRIDE proteomics datasets.

Supplementary Table 2. MS evidence of A-to-I mediated amino acid substitutions.

References

1. Cox,J. and Mann,M. (2008) MaxQuant enables high peptide identification rates, individualized p.p.b.-range mass accuracies and proteome-wide protein quantification. *Nat Biotechnol*, **26**, 1367–1372.
